# Relational Empowerment in Peer‐Mediated Rehabilitation for People With Disabilities: A Constructivist Grounded Theory Study

**DOI:** 10.1111/hex.70966

**Published:** 2026-11-06

**Authors:** Lingyun Liu, Liqiang Cai, Zhuojun Ye, Youyang Tang, Yi Fan, Zhousiqi Tang, Limei Jing

**Affiliations:** ^1^ School of Public Health Shanghai Jiao Tong University School of Medicine Shanghai P.R. China; ^2^ Shuyuan Community Health Service Center of Pudong New Area Shanghai P.R. China; ^3^ School of Public Health Shanghai University of Traditional Chinese Medicine Shanghai P.R. China

**Keywords:** community‐based rehabilitation, constructivist grounded theory, disability, empowerment, peer support

## Abstract

**Background:**

People with disabilities often need rehabilitation support that extends beyond functional recovery to participation, self‐management and community living. We examined how empowerment was generated through peer relationships in China's Self‐help and Peer Support for People with Disabilities (SHPSPD), a peer‐mediated rehabilitation program in the community‐based rehabilitation context.

**Methods:**

Using constructivist grounded theory, we analysed 14 semi‐structured interviews and two focus groups with 24 purposively sampled people with disabilities from SHPSPD. Analysis involved initial and focused coding, constant comparison, memo‐writing, category development and theoretical integration.

**Results:**

The core process was regaining a sense of agency in peer relationships. Participants often began from narrowed life worlds shaped by impairment, stigma, dependence and uncertainty. Entry into peer spaces enabled safe comparison and made change imaginable. Three interrelated mechanisms drove this process: peer mirroring made change believable through embodied evidence of similar others; experiential reciprocity made change actionable through practical knowledge, emotional recognition and resource navigation; and role transformation made change sustainable through helping roles, skill acquisition and participatory engagement. These mechanisms produced outcomes across the intrapersonal, interactional and behavioural dimensions of psychological empowerment and, for some participants, at an emergent community level. This process could be interrupted by limited service reach, accessibility barriers, uneven resources, professional knowledge gaps, cross‐disability boundaries and role burden.

**Conclusions:**

SHPSPD can convert lived disability experience into credible knowledge, practical capacity, participation and contribution when peer spaces are accessible, credible and adequately supported. The findings offer a service‐contextual process explanation of empowerment across psychological and community levels.

**Lived Experience or Public Contribution:**

People with disabilities who participated in SHPSPD were invited to comment on the interpretation of the findings rather than treated only as research participants. Preliminary findings and the emerging theoretical model were shared with them for feedback (member checking), and this feedback was incorporated into the final analysis. The local government assisted with recruitment and access. In this way, the study treated participants' lived experience as a contribution to knowledge.

## 1 Background

Disability is a major public health and rehabilitation concern worldwide. The World Health Organization (WHO) estimates that about 1.3 billion people, or one in six people globally, live with a significant disability and global need for rehabilitation has expanded to the point that at least one in three people would benefit from rehabilitation at some point in the course of illness or injury [1, 2]. Rehabilitation is increasingly understood not merely as treatment of impairments, but as a set of interventions intended to optimise functioning and reduce disability in relation to everyday life [3, 4]. Yet people with disabilities continue to face major inequities in access to health and rehabilitation services, including limited availability, transport barriers, cost, poor continuity of support and weak community‐based follow‐up [5, 6]. These limitations are especially important because rehabilitation needs extend beyond bodily recovery to ongoing activity, participation, self‐management and community living.

In response, disability rehabilitation has increasingly shifted towards community‐based rehabilitation (CBR) and person‐centered and peer‐informed approaches. The WHO CBR Guidelines identify self‐help groups as a core strategy through which people with disabilities can address common problems, enhance strengths, gain skills and improve quality of life [7]. Systematic and scoping reviews suggest that CBR and self‐help or peer‐support approaches may improve functioning, quality of life, social adjustment and access to support, while also offering settings for reciprocity, recognition and social participation [8, 9]. In physical medicine and rehabilitation, peer support has been described as a distinct source of informational, emotional and appraisal support rooted in experiential knowledge, complementing professional care [10]. Theoretical work has framed peer support as relationships of shared responsibility and shared power, through which people move beyond a patient identity toward personal, relational and social change [11]. Evidence suggests peer mentoring during inpatient spinal cord injury rehabilitation has been associated with improved self‐efficacy and fewer unplanned readmissions [12], meta‐analytic evidence among stroke survivors shows benefits for physical and psychosocial outcomes [13], and the credibility of peer mentors has been linked to shared lived experience and relatable communication [14]. Nevertheless, a scoping review of peer‐led interventions found the evidence base to be small and heterogeneous, with most studies examining one‐to‐one mentoring [15]; few studies explain how such support works in everyday rehabilitation trajectories. Conceptual clarity about how peer support is developed, enacted and sustained across disability settings is still lacking.

Empowerment offers a useful lens for understanding these processes. Zimmerman's empowerment theory conceptualises empowerment as both process and outcome, involving increased control over one's life, critical understanding of one's environment and participation in actions that produce meaningful change [16]. It further specifies three interrelated levels of analysis: psychological empowerment (intrapersonal, interactional and behavioural components), organisational empowerment (empowering and empowered organisations) and community empowerment (interrelationships among organisations within a community) [17]. Empowerment has long been valued in disability research and practice, and a body of work has examined a contextual‐behavioral model of empowerment developed through case studies of people with physical disabilities [18], syntheses of strategies for promoting empowerment among individuals with disabilities [19] and studies of disability organisations as empowering settings that challenge stigmatisation and promote emancipation [20], alongside related work in mental health self‐help and qualitative disability research [21, 22]. Relational autonomy theory complements the empowerment perspective by emphasising that agency is not exercised in isolation but through relationships, accessible environments and recognition from others. In disability contexts, this lens reframes independence as self‐directed participation within interdependence rather than self‐sufficiency alone [23, 24].

However, the current literature still shows two important gaps. First, empowerment is often treated as a desirable outcome to be measured, rather than a lived process to be explained; even recent reviews conclude that empowerment research in disability populations remains uneven and conceptually underdeveloped [25]. Second, in China, peer support research has so far been concentrated mainly in mental health and selected community programs, while structured evidence on disability rehabilitation services and empowerment that combine CBR, self‐help groups and peer support remains comparatively limited [26, 27]. This matters because China has recently expanded policy support for disability rehabilitation during the Fourteenth 5‐Year Plan period. Since late 2023, China has promoted a peer‐mediated rehabilitation program for different disability categories called Self‐Help and Peer Support for People with Disabilities (SHPSPD). This service model brings together peer support, skills training, experience sharing, assistive device support and social participation within CBR settings. This creates a need to understand how empowerment is experienced, constructed and constrained within this emerging service context.

In this context, the study examines empowerment pathways among people with disabilities participating in SHPSPD services in China. Guided by constructivist grounded theory and informed by empowerment theory as a sensitising perspective, we aimed to develop a grounded, process‐oriented explanation of empowerment within a community‐based disability rehabilitation service centred on peer relationships. We further focused on participants' accounts of service participation and the relational, organisational and environmental conditions that shaped everyday agency. In doing so, the study contributes to CBR, peer support and disability empowerment literature by explaining how empowerment is generated relationally and conditionally in everyday rehabilitation trajectories.

## 2 Method

### 2.1 Study Design

This qualitative study was conducted as part of a broader research project on SHPSPD services for people with disabilities in China. The present article focuses on how people with disabilities experienced empowerment through participation in SHPSPD, with particular attention to peer relationships, everyday agency and participants' accounts of change in rehabilitation and community life. We adopted constructivist grounded theory to guide data analysis. This approach was appropriate because the study aimed to develop a data‐grounded, process‐oriented interpretation of empowerment as narrated and understood by participants. Following the constructivist perspective, categories were developed through close engagement with interview data, constant comparison, memo‐writing and theoretical integration. We drew on Zimmerman's empowerment theory and relevant disability studies. The study was reported with reference to the Consolidated Criteria for Reporting Qualitative Research (COREQ, see Supplementary file 1 S1) and GRIPP2 checklist (see Supplementary file 1 S3).

### 2.2 Study Setting and Participants

The study was conducted in SHPSPD service settings in a representative eastern province of China. This region has relatively advanced health and social welfare systems and has often served as a pioneer in piloting reforms to improve support for vulnerable groups, including people with disabilities; it formally launched the SHPSPD service in 2024. SHPSPD service refers to a government‐led and peer‐mediated disability rehabilitation service model developed within China's CBR context. The service aims to enhance self‐rehabilitation, social adaptation and community participation through self‐help groups, peer support, skills training, experience sharing, empowerment‐oriented courses, assistive device support and social participation activities. In the study region, SHPSPD service was delivered through rehabilitation institutions, disability service platforms and social organisations.

Participants were initially recruited using purposive sampling, supplemented by snowball referral where appropriate. Potential participants were identified through SHPSPD service platforms, peer mentors and participant referrals. Recruitment sought people with disabilities with direct experience of SHPSPD and variation in participation history and degree of involvement in the service. Through ongoing comparison between cases and iterative refinement of interview probes, recruitment ceased once the main categories reached theoretical sufficiency within the existing dataset [28]. Twenty‐six eligible people with disabilities agreed to be contacted, and 24 ultimately participated: 14 in individual interviews and 10 in two focus group discussions; two did not participate because of privacy concerns or physical condition at the time of data collection.

Inclusion criteria were: aged 18 years or above, holding the Chinese disability certificate, having participated in at least one SHPSPD service activity, and being able to take part in an interview or focus group with or without reasonable communication support. Participants were excluded if they had severe communication barriers that could not be accommodated, acute medical instability or an acute psychiatric crisis that made participation unsafe at the time of data collection. The Chinese disability certificate is an official registration document issued by the China Disabled Persons' Federation and its local branches. Based on the national standard for disability classification and grading (GB/T 26341‐2010), it records the holder's disability category (including visual, hearing, speech, physical, intellectual, mental and multiple disabilities) and severity grade (Grades 1–4, from extremely severe to mild) and serves as the principal eligibility document for accessing disability‐related services and benefits, including rehabilitation programs, assistive device subsidies and welfare support [29]. Our study treated it as a verifiable eligibility criterion.

### 2.3 Data Collection

Data were collected between April and September 2025 through semi‐structured individual interviews and focus group discussions. The interview guideline (see Supplementary file 1 S2) explored participants' pathways into SHPSPD service, experiences of peer interaction, learning from others' lived experience, changes in self‐understanding and daily life, health and rehabilitation self‐management, social participation, helping others, barriers to participation and expectations for future services. The guideline was pilot tested in the first two interviews and refined accordingly.

Four trained researchers (one man and three women) conducted the interviews and focus groups. No researcher had a prior personal relationship with any participant before data collection. Semi‐structured interviews were conducted one‐to‐one, and focus groups were kept small to facilitate participation and interaction among participants with similar service experiences. No service staff or other non‐participants were present during interviews or focus groups. Data collection took place in quiet, accessible settings convenient to participants; when needed, interviews were conducted using a secure online format or by telephone. Interviews and focus groups were conducted in Chinese. Sessions lasted approximately 35–90 min and were audio‐recorded with permission. Participants received a handheld fan and soap as a token of appreciation. Field notes were written during or immediately after data collection to record contextual information, non‐verbal cues when observable, early analytic impressions and reflections on the interview process. Recordings were transcribed verbatim in Chinese and checked for accuracy by trained members of the research team. Transcripts were returned to participants for comment or correction. Identifying information was removed or replaced with participant codes before analysis. Analysis was conducted primarily in Chinese; selected quotations were translated into English for reporting and checked by bilingual researchers to preserve meaning.

### 2.4 Data Analysis

Data analysis followed Charmaz's constructivist grounded theory approach [30]. Initial coding was conducted by two researchers independently for a subset of transcripts to establish a shared coding approach; subsequent coding was conducted by the lead author, with regular team discussions to resolve differences through consensus. The transcripts were managed in Excel and NVivo 15.0, which was used to record transcript segments, codes, analytic memos, category development and links between quotations and their original locations. Analysis began with repeated reading of the transcripts to become familiar with participants' accounts. Initial coding was then conducted line by line or segment by segment. Codes were kept close to the data and were written, where possible, in action‐oriented terms to capture processes, interactions and changes described by participants. Focused coding was then used to identify the most frequent, significant or analytically useful initial codes. Through this process, related codes were compared, grouped, refined or separated. Constant comparison was used throughout the analysis, including comparisons between interview segments, between participants, between individual interviews and focus group discussions, and between emerging codes and possible negative cases.

Memo‐writing accompanied all stages of analysis. Memos were used to record coding decisions, compare cases, clarify category boundaries, note uncertainties and develop possible relationships among emerging categories. As analysis progressed, focused codes were developed into more abstract categories by examining their properties, dimensions, conditions and consequences. Theoretical integration was then used to examine how categories related to one another and to construct a process‐oriented explanation of empowerment in SHPSPD service. Zimmerman's empowerment theory and disability studies literature were used as sensitising perspectives during interpretation, but they were not used as predetermined coding categories. Categories were developed inductively from the interview and focus group data and were then considered in relation to existing theoretical concepts.

### 2.5 Research Team, Reflexivity and Rigour

All members of the research team held graduate degrees and backgrounds in public health, rehabilitation service research and disability‐related health services. None was the direct service provider for the participants. Before and during data collection and analysis, the team reflected on and documented how prior familiarity with disability rehabilitation services and assumptions about empowerment, peer support and service value might shape interviewing and interpretation. Participants were told that critical or negative experiences were as important as positive accounts. Four participants returned feedback on the preliminary categories and the emerging model. Reflexive notes were used to record early impressions, methodological decisions and potential assumptions.

Trustworthiness was strengthened through several strategies. Credibility was supported by iterative data collection and analysis, constant comparison, memo‐writing, team discussion and attention to negative and ambivalent cases. Dependability was supported by an audit trail documenting coding decisions, category development and changes to the emerging theoretical model. Confirmability was enhanced by returning repeatedly to original transcript segments when refining categories. Transferability was supported through description of the service setting, participant roles, sampling process and analytic procedures. The analytic procedure, coding framework, negative cases and key methodological decisions are summarised in Supplementary file 2.

## 3 Results

### 3.1 Participant Characteristics and Theoretical Model

Twenty‐four people with disabilities participated in the study. Participants varied in gender, age, years with disability, degree and type of disability (Table 1). Illustrative quotations are identified by anonymised participant numbers (P01–P24).

**Table 1:** Participant characteristics.

Characteristic		Frequency (*n*)	%
Gender	Female	14	58
	Male	10	42
Age	18–44 years	9	38
	45–64 years	10	42
	> 65 years	5	21
Years in disability	< 5 years	5	21
	5–10 years	3	13
	> 10 years	16	67
Degree of disability†	Grade 1	15	63
	Grade 2	6	25
	Grade 3	3	13
	Grade 4	0	0
Type of disability‡	Physical disability	16	67
	Visual disability	2	8
	Hearing disability	2	8
	Mental disability	2	8
	Multiple disabilities	2	8
Role in SHPSPD service	Participant	15	63
	Peer volunteer	5	21
	Peer mentor	4	17

^†^ Grade 1 = extremely severe, Grade 2 = severe, Grade 3 = moderate, and Grade 4 = mild.

^‡^ Disability types were categorised according to the Chinese disability certificate.

*Note:* Percentages are rounded and may not sum to 100.

Analysis generated a grounded theory of regaining a sense of agency in peer relationships within the SHPSPD service in a CBR context: from receiving help to reciprocal helping. Seven interrelated categories constituted this process. Six formed the core process: disempowerment as a starting point, entering peer spaces, peer mirroring and safe spaces, experiential reciprocity, role transformation, and interruptions and reversals in the empowerment process. The seventh, public expression and service demands, reflected early forms of service‐oriented voice, but the data did not support interpreting this as organised collective advocacy. Three interrelated empowerment mechanisms operated across these categories: the reflective mechanism (peer mirroring), the relational mechanism (experiential reciprocity) and the active mechanism (role transformation). These mechanisms produced outcomes at psychological and emergent community levels consistent with Zimmerman's multilevel framework. The model was not a fixed sequence. Empowerment was conditional, relational and sometimes reversible. Figure 1 summarizes the grounded theoretical model, and Table 2 maps the empowerment mechanisms onto Zimmerman's framework.

**Figure 1:**
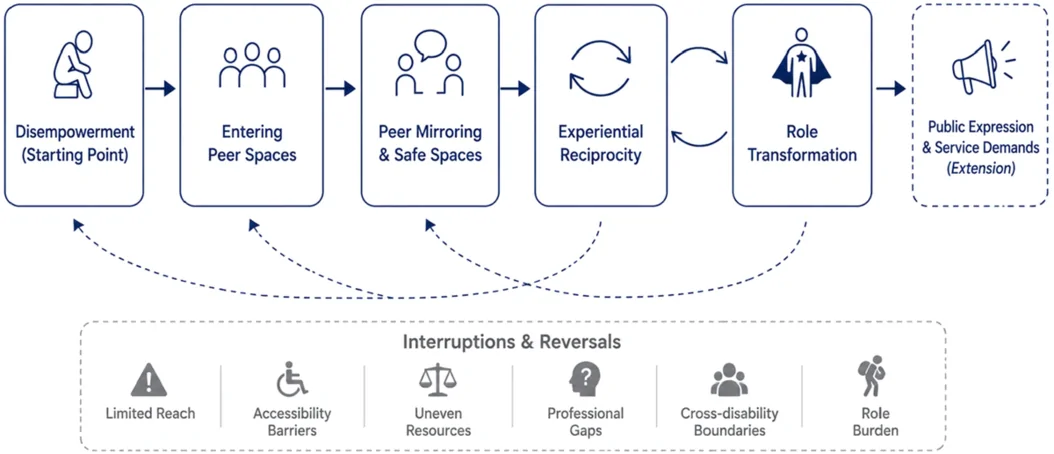
Grounded theoretical model of regaining a sense of agency in peer relationships.

**Table 2:** Mapping of empowerment processes onto Zimmerman's framework.

Empowerment mechanism	Core function	Zimmerman's level	Grounded components	Representative quotation
**Reflective (Peer mirroring and recognition)**	Making change believable	Psychological (intrapersonal)	Perceived control; self‐efficacy; identity reconstruction; meaning remaking; future imagination	‘I saw that she was so young and beautiful, and even though she used a wheelchair, she could still be happy. Then I thought, why can't I?’ (P01)
**Relational (Experiential reciprocity)**	Making change actionable	Psychological (interactional)	Critical awareness; resource navigation; reciprocal relationships; relational reconstruction; instrumental mutual aid	‘Rehabilitation knowledge is something we pass on to one another. For example, I share rehabilitation policies I've heard about with others, and they share the wheelchair techniques they've learned with me’ (P20)
**Active (Role transformation)**	Making change sustainable	Psychological (behavioural); community (emergent)	Role action; skill acquisition; participatory decision‐making; mutual healing; service voice	‘Later I wanted to help more people and became an assistant teacher, using my own feelings and experience to help those who had not yet come out’ (P01)

*Note:* The three mechanisms operate in parallel and interact, rather than following a fixed sequence. Outcomes at each level may reinforce earlier mechanisms through feedback loops.

### 3.2 Disempowerment as a Starting Point: Shrinking Life Worlds and Fractured Self‐Understanding

Participants described the period before entering SHPSPD service as a time when disability narrowed their life world. Disempowerment was produced through bodily limitation, social gaze, dependence in daily life, loss of ordinary roles and limited access to credible rehabilitation knowledge. For some, the home, hospital ward or bed became a protective but confining space. One participant described several years after injury as dominated by stigma and withdrawal:

I went through a long period of stigma, about five or 6 years. I could not accept other people's eyes when I went out.

P07

The same participant described home and the ward as the safest spaces, but this safety also confirmed his confinement:

When people asked about me, I felt afraid. I almost wanted to go out wrapped in a quilt, or simply not go out. I just wanted to hide at home and in the hospital ward.

P07

For participants with severe physical impairment, disempowerment was also rooted in dependence in daily bodily management. A participant with high‐level SCI described the practical intensity of everyday life:

My self‐care ability is very poor. I need someone almost 24 h a day. Managing bowel and bladder problems is extremely inconvenient, and quality of life is very poor.

P12

The starting point of empowerment was therefore not neutral. Participants were often living within restricted bodily, emotional and social conditions that limited action and future imagination. However, a negative case showed that delayed participation did not always reflect stigma or lack of resources. One participant refused training for several years because he believed he already possessed the skills being offered.

They called me to join the training class for two or 3 years, but I did not want to go. I asked what I would do there. They said I would practice transfer skills and other things. I said I already knew all of that and did not need to learn it.

P03

This case suggests that disempowerment was not a single condition. For some, the barrier was shame or fear; for others, it was mismatch between perceived needs and available services, personal habit, or limited readiness to engage. The pathway into empowerment therefore began from different forms of constrained agency.

### 3.3 Entering Peer Spaces: Referral, Being Brought Out and Gradual Participation

Participants rarely entered SHPSPD service through purely individual initiative. Entry was often mediated by rehabilitation therapists, disability service platforms, peers or home visits. The first step was therefore not simply joining an activity, but being connected to a peer space that made participation imaginable. For one participant who had resisted repeated invitations, attending once became a turning point:

Later I thought it through. Since they came every year, I went once, just to play and exercise. After I went, it was the first time I saw so many wheelchair users together. After that, I joined the art troupe.

P03

Another participant framed the platform as a space designed to bring people out of the home and back toward society:

It was a platform for people with SCI. The idea was self‐help and mutual help among people with SCI, helping disabled people who had been staying at home to come out, move toward society, and use rehabilitation to get out of the home.

P12

Entry into peer spaces was often gradual. Some participants first observed, resisted or attended occasionally before becoming more involved. This category therefore captured both access and orientation: participants had to find, or be brought into, a space where disability became a shared condition around which learning, comparison and mutual support could occur.

### 3.4 Peer Mirroring and Safe Spaces: Seeing Possibility Through Similar Others

A central mechanism was peer mirroring. Participants described being changed by seeing others with similar impairments living actively, confidently or socially. These encounters provided embodied evidence that professional reassurance alone could not provide. One participant described meeting a young wheelchair user who appeared joyful and capable:

I saw that she was so young and beautiful, and even though she used a wheelchair, she could still be happy. Then I thought, why can't I? It was like seeing a beam of light in a swamp. Whether I could catch it depended on me, and I caught that light and came out.

P01

Another participant recalled seeing older peers singing and enjoying life during training. Their example gradually opened another way of interpreting life after injury:

They loved life. After meals they would sing. At that time, I wondered how they could do that. I could not understand it then. Seeing them made me feel that this training class gave people a different understanding. From helplessness and depression, I saw hope.

P02

Peer mirroring was powerful because peers were perceived as credible. Participants contrasted demonstration by disabled peers with instruction from non‐disabled professionals. A peer mentor explained that similar bodily experience made teaching more convincing:

If a teacher who can use their legs demonstrates, others may say, ‘You are normal, but I cannot do that.’ But I also use a wheelchair, and my injury level may be similar to yours. If I demonstrate that I can do it, then under normal circumstances you should also be able to. When we do it, it is more convincing.

P03

This credibility was not only technical. Disabled peer teachers made successful action appear embodied, socially believable and transferable because they taught from within a similar bodily position. Peer spaces also functioned as safe spaces. In one focus group, a participant described the peer setting as a place where confidence could be temporarily restored, even if everyday society remained difficult:

When I was there, I felt I could do it. But after going back and facing reality, I might not feel so confident. Outside, there is discrimination and many worries. So, every time there is an activity, I take part. It feels like returning to recharge myself.

P04

This recharging did not eliminate stigma or environmental barriers. It created relational conditions in which participants could imagine themselves as capable before testing that capability in wider life. The strength of peer mirroring depended on perceived similarity: the most powerful role models were those whose impairment, functional limitations or life stage felt close enough to make success believable. Perceived similarity worked as a condition of transfer: participants could move from ‘that person can do it’ to ‘perhaps I can try’.

Beyond seeing possibility, peer mirroring also contributed to meaning remaking. Several participants described a shift from understanding disability as a burden or punishment toward reconstructing a meaningful life narrative. This shift was closely tied to peer examples: seeing others who had found purpose within disability made such purpose appear achievable. As one participant reflected:

Now I feel that although I have a disability, life still has meaning.

P02

### 3.5 Experiential Reciprocity: Translating Lived Knowledge Into Practical Capacity

Within SHPSPD service, mutual aid was not limited to emotional support. Participants described a circulation of practical knowledge that helped them manage bodily care, assistive devices, mobility, rehabilitation and service information. Lived experience became useful when it was shared, adapted, practised and sometimes passed on to others.

Rehabilitation knowledge is something we pass on to one another. For example, I share rehabilitation policies I've heard about with others, and they share the wheelchair techniques they've learned with me. That is also a kind of mutual aid.

P20

For many participants with physical disabilities, capacity depended on both professional rehabilitation knowledge and experiential translation. Yet formal rehabilitation knowledge became most useful when translated into ordinary life by people with similar experience. One participant described how catheterisation training became a resource for mutual aid after she returned to her local peer network:

They told us that peers, especially those with rehabilitation knowledge, should help each other. For example, many people ask how to do catheterization. At this point, professional medical advice isn't particularly helpful, whereas those of us with long‐term experience often know more.

P05

Experiential reciprocity further involved translating rehabilitation skills into unpredictable public environments:

At the rehabilitation center there may be only one or two curbs, but conditions on the road are different. When you go out, you meet people and have to ask for help. Some things have to be faced and solved by yourself.

P03

For participants with sensory disabilities, experiential knowledge centred less on bodily transfer and more on communication control and information access. Skills such as speech‐to‐text tools, emergency calls, mobile phone functions and disability‐specific services became means of regaining autonomy in everyday interaction. Across disability types, the common mechanism was not the content of the skill itself, but the transformation of lived experience into usable capacity.

Experiential reciprocity also extended beyond face‐to‐face settings through digital peer networks. Participants described using online groups to share problems, exchange information and maintain contact between in‐person activities:

We have a dedicated WeChat group. Whenever someone encounters a problem, they raise it in the group, and everyone discusses it together.

P16

These digital networks sustained experiential knowledge sharing between formal sessions.

A peer who also has paraplegia developed a pressure sore, so we all learned that we must develop habits to avoid this. These things you only learn if you come here; the skills can be applied to daily life.

P16

Beyond practical knowledge, experiential reciprocity contributed to relational reconstruction.

My relationship with my husband has improved a lot, because I am no longer so sensitive.

P02

Another described shifting family power dynamics:

My family has started to respect my opinions; before, they decided everything for me.

P01

When my family saw my changes, they also supported me in continuing to come.

P05

However, participants did not romanticise experiential knowledge as automatically reliable. Peer experience and service organisation required professional calibration. One participant questioned the professional capacity of some service staff:

There may be one or two social workers, but basically there are not many people, and there is little professionalism. They do not even know how to disassemble our wheelchairs. It is more like just sending messages in a group.

P01

These negative cases indicate that experiential reciprocity was empowering when it was credible, practical and supported by appropriate professional knowledge. Without such support, mutual aid risked becoming incomplete, inaccurate or uneven.

### 3.6 Role Transformation: From Receiving Help to Helping Others

For some participants, empowerment became most visible when they moved from receiving help to helping others. This role transformation was not simply volunteering. It represented the conversion of disability experience into a resource that could guide, teach, encourage or protect others. One participant connected his own process of being helped to his later desire to support newcomers:

Later I wanted to help more people and became an assistant teacher, using my own feelings and experience to help those who had not yet come out but wanted to come out, so that they could take fewer detours.

P01

Role transformation also produced mutual healing—a reciprocal benefit in which helping others simultaneously strengthened the helper. As one peer mentor observed:

I set a goal for myself, and I have already reached the goal I set for myself. Helping yourself—that gives you positive feedback.

P16

It also involved participatory engagement in service decisions, which contributed to a sense of ownership over the service process.

Some activities were proposed by us, and the center adopted them.

P05

Still, role transformation was not universal. Some participants remained primarily service users rather than taking on helping roles and experienced empowerment as stable participation, greater confidence in going out, better self‐management or a less isolated life.

After I kept going, living on my own became much better. I had to think things through and become more self‐disciplined. That gave me a sense of achievement and helped my confidence. Now I basically do not see myself only as a disabled person; I just live my life normally.

P09

Helping others could also create new burdens. A participant with teaching experience described the physical and emotional demands of supporting many trainees while managing his own disability:

After injury, daily life is already very tiring. If you become a teacher and take care of students, each student only has to focus on themselves, but as a teacher I have to face 15 or 16 students in a day. I am very tired.

P01

Thus, helping others was both an empowering outcome and a potential source of exhaustion. Sustaining peer helper roles required organisational support, realistic workloads and recognition of disabled peer mentors' own bodily limits.

### 3.7 Interruptions and Reversals: Conditions That Shaped the Empowerment Process

The empowerment process was described as conditional. It could be delayed before entry, narrowed during participation or strained after role transformation. Barriers included limited service reach, accessibility barriers, uneven resources, professional knowledge gaps, cross‐disability boundaries and role burden.

First, services often reached people who were already connected to peer networks while missing those who were most isolated. Participants therefore emphasised outreach beyond closed peer circles:

I think publicity should be increased; that is necessary. I also hope to add home visits. We could regularly visit injured peers through communities or towns. When the visitor is also an injured peer, they are persuasive. They can say, look at this environment and how we came out through this platform. Some participants really registered through this channel.

P04

Second, environmental and policy conditions shaped whether participation could be sustained. One participant described the ambiguity of modified mobility devices and the distance between home and public transport:

The wheelchair can be connected to a motorized front attachment, but then there is a problem: this transport tool is not legal. That is a big issue. Also, it depends on the distance to the metro station. If it is far from the station, it is inconvenient.

P10

Service resources and administrative context also mattered. One participant described how opportunities changed after local administrative adjustment:

Before, when we applied to go to a rehabilitation center, the previous district did not allow it and said they would not take responsibility if something happened during activities. After the adjustment, conditions became better and it was different.

P13

Professional knowledge gaps could interrupt experiential reciprocity. Peer support was powerful when grounded in lived experience, but insufficient when staff or peers lacked technical knowledge. Shared experience could also become problematic when outdated or partial knowledge circulated without professional correction.

Finally, peer similarity had limits. Cross‐disability mutual aid did not always produce recognition. One participant described the difficulty of relating across disability categories:

It is honestly difficult across groups. For example, people who are deaf, blind, or use wheelchairs are all disabled, but we are different. Sometimes there is exclusion, not because we want to exclude others, but because our functions are different.

P01

This account qualifies the idea of peer support. Similarity was not a background detail; it was part of the mechanism. When experiences were too different, participants needed additional translation, mediation or design support for mutual aid to work across disability categories. Together, these interruptions show that empowerment depended on whether peer spaces were reachable, credible, inclusive, professionally supported and usable in everyday environments.

### 3.8 Public Expression and Service Demands as a Tentative Extension

Public expression and service demands appeared as a tentative extension of empowerment. Some participants moved beyond personal change to articulate service‐oriented needs concerning publicity, accessibility, assistive devices, policy information and service design. One participant described using short videos to share positive experiences with peers who had concerns:

I recently filmed small videos of my daily life and sent them to friends who had concerns. Short videos are very convenient now. By sharing positive things and our own experiences, I think it can help some friends.

P04

Another participant called for clearer information about assistive device services:

For assistive device applications, honestly, I do not even know what can be applied for. It is too confusing. I hope the catalogue can clearly tell us each year what services the government has purchased.

P01

These accounts suggested an emerging public voice, but the data did not show sustained organising, shared agendas or direct policy influence.

## 4 Discussion

This constructivist grounded theory study developed a process‐oriented explanation of empowerment in SHPSPD service settings among people with disabilities. The core process was regaining agency through peer relationships, moving unevenly from being helped to becoming able, in some cases, to help others. Empowerment was not experienced as a single psychological outcome or as a direct effect of service exposure. Rather, it was constructed through entry into peer spaces, recognition of similar others as credible examples, circulation of lived and practical knowledge, and gradual role transformation. At the same time, this process was conditional. Limited service reach, accessibility barriers, uneven resources, professional knowledge gaps, cross‐disability boundaries and role burden could interrupt or redirect empowerment. These findings suggest that peer‐mediated rehabilitation settings can convert disability experience into knowledge, participation and social contribution when they are sufficiently accessible, credible and supported.

### 4.1 Interpreting Empowerment as a Relational, Multilevel Process

Our findings support an understanding of empowerment as a process rather than a static attribute, which is consistent with the perspective of empowerment theory [17, 31]. Our study adds empirical detail to this process in disability rehabilitation. Participants did not simply become “more empowered” after receiving support. They first encountered conditions that made action imaginable. These included a peer space, a similar body, a credible example, a practical technique or a role in which their own experience could matter to someone else. This finding is consistent with Lord and Hutchison's emphasis on empowerment as a dynamic process shaped by life events, supportive relationships, opportunities for action and changes in self‐understanding [32]. In this study, peer spaces functioned as opportunity structures. They did not give participants agency from outside; rather, they made existing and emerging capacities visible, usable and socially recognised. Seeing similar others living, participating, teaching or helping challenged participants' earlier assumptions about disability and possibility. This mechanism is more specific than general social support. It involved embodied credibility: the person offering encouragement or instruction was not merely sympathetic, but was perceived as someone whose experience made their advice believable.

The three mechanisms identified in this study correspond broadly to Zimmerman's multilevel framework. The reflective mechanism (peer mirroring and recognition) primarily produced psychological‐level outcomes: participants reconstructed identity, remade meaning and developed self‐efficacy through seeing credible others. The relational mechanism (experiential reciprocity) produced interactional outcomes: participants navigated resources, rebuilt family and peer relationships and developed critical awareness of service systems. The active mechanism (role transformation) extended into behavioural and emergent community‐level outcomes: participants acquired skills, took on helping roles and began to voice service needs. This correspondence is not a simple mapping. The mechanisms operated in parallel and interacted, and outcomes at one level could reinforce processes at another through feedback loops.

Positioned in relation to this literature, the present study extends rather than replaces earlier disability empowerment research [18, 19, 20]. Our findings add to disability empowerment work in four ways: first, we specify the relational micro‐mechanisms of peer mirroring, experiential reciprocity and role transformation through which empowerment is generated within a rehabilitation service rather than an advocacy or organizational setting; second, we show that these mechanisms are conditional and can be interrupted by limited service reach, accessibility barriers, gaps in professional support and the limits of peer similarity; third, we extend disability empowerment research to community‐based rehabilitation in China, a middle‐income country context, complementing a literature developed mainly in Western settings; fourth, we also clarify the link between psychological empowerment and participation. Participants' confidence was grounded in practice. Thus, empowerment was not only a change in self‐perception. It was a change in what participants could try, where they could go, whose knowledge counted and what roles they could occupy.

### 4.2 Peer Support as Embodied Evidence and Experiential Reciprocity

Recent rehabilitation literature has increasingly described peer support as more than informal encouragement [33, 34]. Magasi and Papadimitriou argue that the field has accumulated evidence of development and outcomes but still needs greater conceptual clarity about how peer support interventions are structured, theorised and evaluated [10]. Our research addresses this question by distinguishing and exploring two mechanisms in peer support: peer mirroring and experiential reciprocity. While Bandura's social learning theory identifies modelling as a general mechanism of behavioural acquisition [35], peer mirroring in our study added an embodied, disability‐specific dimension: the credibility of the model depended on sharing a similar bodily condition. Experiential reciprocity refers to the circulation of practical knowledge, emotional recognition and resource navigation among peers. In our data, these mechanisms worked together: peer mirroring made change believable, while experiential reciprocity made change actionable. This helps explain why peer support could do more than provide companionship or encouragement and extends Borkman's concept of experiential knowledge [36]. It converted lived disability experience into credible evidence, practical capacity and, in some cases, a basis for helping others.

This does not mean that peer experience should replace professional knowledge. A key contribution of the negative cases is that they prevent an overly romantic account of mutual aid. Some participants questioned whether peers or staff had sufficient technical knowledge, and some accounts cautioned that individual experience could be outdated or not transferable. Experiential knowledge was empowering when it was practical, credible and open to professional calibration. This point is important for rehabilitation systems: peer support is strongest when positioned as a complementary form of knowledge, not as a low‐cost substitute for professional rehabilitation, assistive technology expertise, psychological support or case coordination.

Our findings also resonate with research on self‐help groups in rehabilitation. A Norwegian study found that self‐help groups in psychosocial rehabilitation supported learning and practising safely, provided refuge from external expectations and helped participants strengthen positive inner processes [9]. The present study extends these findings by showing how safe learning environments worked through disability‐specific credibility and peer comparison and by identifying role transformation as a later process through which participants' own experience became a resource for others.

### 4.3 From Receiving Help to Helping Others: Relational Autonomy and Mutual Healing

The transition from receiving help to helping others was central to our study. This transition should not be interpreted narrowly as volunteering. It marked a change in social position: participants' disability experience became knowledge that could guide, protect, reassure or teach others. Participants became empowered not only by gaining confidence, but by being recognised as credible actors within a peer network.

This role transformation also helps refine the meaning of self‐help in disability rehabilitation. Self‐help did not mean becoming independent in an individualistic sense. Disability studies and independent living scholarship have long challenged the idea that independence means doing everything alone, instead emphasising choice, control and the ability to direct one's own life and support [37, 38]. In our study, many participants continued to require interpersonal, professional, organisational and environmental support. Self‐help therefore meant regaining a capacity to act within interdependence: to manage one's body more effectively, move through public space, ask for resources, reinterpret disability and contribute to others. This interpretation is consistent with relational autonomy and care scholarship. This perspective emphasises that agency is made possible through accessible environments, credible peer examples, practical knowledge and recognition from others. At the same time, environmental barriers and supports shape whether participation can be enacted in everyday life [23, 24, 39]. In this framework, mutual healing is an integral feature of relational empowerment.

However, the helper role also created tension. Peer mentors, teachers and group leaders could experience fatigue, responsibility pressure and insufficient support. This finding echoes a broader concern in empowerment practice: shifting from professional dominance to peer participation should not become a transfer of responsibility from institutions to disabled individuals without resources [40, 41, 42]. Peer helper roles require training, supervision, role clarity, reasonable workload, emotional support and recognition. Otherwise, empowerment may become uneven: participants who help others may gain meaning and status, but also absorb burdens that the service system has not adequately supported.

### 4.4 Public Expression as an Emerging but Limited Extension

Some participants moved from personal experience toward service‐oriented critique, suggesting improvements in publicity, assistive device information, accessibility and service design. This finding indicates an emerging public voice, but the evidence did not support strong claims of organised collective advocacy. This distinction matters. Empowerment theory distinguishes organisational and community levels of analysis, but not every expression of need is collective action. In this study, public expression appeared as a tentative extension of role transformation: participants began to use their experience to identify what would help others, but the data did not show sustained organising, shared agendas or policy influence.

This cautious interpretation strengthens rather than weakens the analysis. It shows that empowerment may begin with personal and relational change before becoming public or collective. For services, the implication is that platforms should not assume advocacy will emerge automatically from participation. If service voice is desired, it needs structures: regular feedback channels, participant advisory roles, peer leader development, accessible information about policies and assistive devices, and mechanisms through which suggestions can be answered. Without such structures, public expression may remain individual, episodic and dependent on particularly articulate participants.

### 4.5 Implications

Several implications follow from the findings. First, SHPSPD service should be designed as a staged pathway rather than a set of isolated activities. Participants may need different forms of support at different points: outreach and invitation before entry, safe observation and peer matching during early participation, practical skills and resource navigation during engagement, and role support for those who become peer helpers.

Second, peer matching should be treated as a substantive design issue. Similarity in disability type, life stage, injury timing, communication mode or participation goal may make peer examples more credible. At the same time, services should avoid creating closed circles that exclude other disability groups. Cross‐disability mutual aid requires facilitation, translation and attention to communication and bodily differences.

Third, experiential knowledge should be formally valued but professionally supported. Services can build mechanisms for peer mentors to share lived knowledge while also ensuring access to rehabilitation professionals, assistive technology expertise, psychological support and safeguarding procedures. This balance avoids both professional dominance and unsupported peer burden.

Fourth, empowerment‐oriented services should address environmental and organisational conditions. The service platform should connect peer support with accessibility advocacy, resource navigation, supported communication and service feedback systems.

## 5 Strengths and Limitations

This study has several strengths. It used constructivist grounded theory to move beyond describing service experiences and to develop a process‐oriented explanation of empowerment. The analysis drew on individual interviews and focus groups, included participants with multiple disability types and roles, and retained negative cases to avoid an overly positive account of self‐help and mutual aid. The resulting model is grounded in participant accounts while engaging with empowerment theory and disability rehabilitation literature.

Several limitations should also be acknowledged. First, the sample included participants who had already entered SHPSPD service. People who remained isolated, declined participation or withdrew after negative experiences were less visible in the data. Their experiences may have further clarified why empowerment does not occur. Second, although the sample included different disability groups, some voices were stronger than others, particularly those of physically disabled participants and peer leaders. Due to difficulties in expression, individuals with intellectual or speech disabilities were not included in this study, which will be addressed in future research. Third, although the main theoretical categories reached theoretical sufficiency, the study was conducted in one developed eastern region of China; transferability to regions with different service capacity, accessibility conditions and community resources should be assessed cautiously. Fourth, the study did not directly assess organisational empowerment (e.g., resource acquisition, network development or policy influence). Organisational conditions such as service reach, professional support and recognition of peer helper roles were captured through participants' accounts and analysed as facilitators or barriers, not as organisational outcomes. The mapping onto Zimmerman's framework is therefore interpretive and adapted to the service context rather than a formal test of the framework.

## 6 Conclusion

This study shows that empowerment in SHPSPD service is generated through a relational process of regaining agency. Participants were not empowered simply by receiving services. They were brought into peer spaces, saw possible selves through credible others, translated lived experience into practical capacity and, in some cases, became resources for others. Three interrelated mechanisms drove this process, producing outcomes at psychological and emergent community levels. This process reframes disability rehabilitation as more than functional recovery or service utilisation. It highlights the value of peer relationships as settings where knowledge, confidence, participation and contribution can be reconstructed. At the same time, empowerment remained conditional on service reach, accessibility, resource equity, professional support, cross‐disability fit and recognition of peer helper roles. Self‐help and mutual aid can therefore contribute to disability empowerment, but only when supported as a relational, organisational and community process rather than left to individual resilience alone.

## Author Contributions

**Lingyun Liu:** formal analysis, methodology, visualisation, writing – original draft, software, data curation, validation. **Liqiang Cai:** writing – review and editing, supervision, funding acquisition. **Zhuojun Ye:** conceptualisation, methodology, project administration, supervision, validation. **Youyang Tang:** investigation, data curation. **Yi Fan:** investigation, supervision. **Zhousiqi Tang:** investigation. **Limei Jing:** conceptualisation, resources, funding acquisition, writing – review and editing, project administration, supervision.

## Funding

The authors have nothing to report.

## Ethics Statement

This study was approved by the ethics committee of the Shanghai Jiao Tong University School of Medicine (ref: SJUPN‐2025‐020‐KS1). All study procedures were carried out in accordance with the Declaration of Helsinki. All participants were informed about the purpose of the study, assured of confidentiality and provided written consent prior to participation. Participation was voluntary, and respondents could withdraw at any time without consequence.

## Consent

All participants were informed about the purpose of the study, assured of confidentiality and provided written consent prior to participation. Participation was voluntary, and respondents could withdraw at any time without consequence.

## Conflicts of Interest

The authors declare no conflicts of interest.

## Reflexivity Statement

The research team consists of public health scholars and rehabilitation professionals. Throughout the research process, the authors maintained a reflexive stance regarding their positions as academic researchers and their potential influence on data interpretation. None of the authors was the direct service provider for people with disabilities.

## Supporting information


Supporting File 1

Supporting File 2

## Data Availability

The qualitative interview and focus group data generated and analysed during the current study are not publicly available because they contain potentially identifiable and sensitive personal information from participants with disabilities. Public sharing would compromise participant confidentiality and violate the conditions of ethical approval and informed consent. De‐identified excerpts supporting the findings are included in the article and supplementary file. Additional information may be available from the corresponding author upon reasonable request and subject to ethical approval.

## References

[1] World Health Organization , 10 Facts on Disability (World Health Organization, 2023), https://www.who.int/news-room/facts-in-pictures/detail/disabilities.

[2] A. Cieza , K. Causey , K. Kamenov , S. W. Hanson , S. Chatterji , and T. Vos , “Global Estimates of the Need for Rehabilitation Based on the Global Burden of Disease Study 2019: A Systematic Analysis for the Global Burden of Disease Study 2019,” Lancet 396, no. 10267 (2021): 2006–2017, 10.1016/S0140-6736(20)32340-0. PMC7811204 33275908

[3] World Health Organization , Rehabilitation in Health Systems (World Health Organization, 2017), https://www.ncbi.nlm.nih.gov/books/NBK552492/.

[4] World Health Organization , Rehabilitation 2030 Initiative (World Health Organization, n.d.), https://www.who.int/initiatives/rehabilitation-2030.

[5] T. Bright , S. Wallace , and H. Kuper , “A Systematic Review of Access to Rehabilitation for People With Disabilities in Low‐ and Middle‐Income Countries,” International Journal of Environmental Research and Public Health 15, no. 10 (2018): 2165, 10.3390/ijerph15102165. PMC6210163 30279358

[6] X. Hunt , M. Bradshaw , S. L. Vogel , et al., “Community Support for Persons With Disabilities in Low‐ and Middle‐Income Countries: A Scoping Review,” International Journal of Environmental Research and Public Health 19, no. 14 (2022): 8269, 10.3390/ijerph19148269. PMC9319493 35886121

[7] World Health Organization, International Labour Organization, United Nations Educational, Scientific and Cultural Organization, International Disability and Development Consortium , Community‐Based Rehabilitation: CBR Guidelines (Geneva: World Health Organization, 2010), https://iris.who.int/handle/10665/44405.

[8] V. Iemmi , L. Gibson , K. Blanchet , et al., “Community‐Based Rehabilitation for People With Disabilities in Low‐ and Middle‐Income Countries: A Systematic Review,” Campbell Systematic Reviews 11, no. 1 (2015): 1–177, 10.4073/CSR.2015.15.

[9] B. J. Landstad , M. Hedlund , and E. Kendall , “Practicing in a Person‐Centred Environment—Self‐Help Groups in Psycho‐Social Rehabilitation,” Disability and Rehabilitation 44, no. 7 (2022): 1067–1076, 10.1080/09638288.2020.1789897. 32673133

[10] S. Magasi and C. Papadimitriou , “Peer Support Interventions in Physical Medicine and Rehabilitation: A Framework to Advance the Field,” supplement, Archives of Physical Medicine and Rehabilitation 103, no. 7 Suppl (2022): S222–S229, 10.1016/j.apmr.2020.09.400. 33440133

[11] S. Mead , D. Hilton , and L. Curtis , “Peer Support: A Theoretical Perspective,” Psychiatric Rehabilitation Journal 25, no. 2 (2001): 134–141, 10.1037/h0095032. 11769979

[12] M. L. Jones , J. Gassaway , and W. M. Sweatman , “Peer Mentoring Reduces Unplanned Readmissions and Improves Self‐Efficacy Following Inpatient Rehabilitation for Individuals With Spinal Cord Injury,” Journal of Spinal Cord Medicine 44, no. 3 (2021): 383–391, 10.1080/10790268.2019.1645407. PMC8081317 31403374

[13] X. Wan , J. P. C. Chau , H. Mou , and X. Liu , “Effects of Peer Support Interventions on Physical and Psychosocial Outcomes Among Stroke Survivors: A Systematic Review and Meta‐Analysis,” International Journal of Nursing Studies 121 (2021): 104001, 10.1016/j.ijnurstu.2021.104001. 34246069

[14] H. L. Gainforth , E. E. Giroux , R. B. Shaw , et al., “Investigating Characteristics of Quality Peer Mentors With Spinal Cord Injury,” Archives of Physical Medicine and Rehabilitation 100, no. 10 (2019): 1916–1923, 10.1016/j.apmr.2019.04.019. 31153855

[15] L. Barclay and G. M. Hilton , “A Scoping Review of Peer‐Led Interventions Following Spinal Cord Injury,” Spinal Cord 57, no. 8 (2019): 626–635, 10.1038/s41393-019-0297-x. 31123333

[16] D. D. Perkins and M. A. Zimmerman , “Empowerment Theory, Research, and Application,” American Journal of Community Psychology 23, no. 5 (1995): 569–579, 10.1007/BF02506982. 8851340

[17] M. A. Zimmerman , “Empowerment Theory: Psychological, Organizational and Community Levels of Analysis,” in Handbook of Community Psychology, ed. J. Rappaport and E. Seidman (Springer, 2000), 43–63, 10.1007/978-1-4615-4193-6_2.

[18] S. B. Fawcett , G. W. White , F. E. Balcazar , et al., “A Contextual‐Behavioral Model of Empowerment: Case Studies Involving People With Physical Disabilities,” American Journal of Community Psychology 22, no. 4 (1994): 471–496, 10.1007/BF02506890. 7754999

[19] F. E. Balcazar and Y. Suarez‐Balcazar , “Promoting Empowerment Among Individuals With Disabilities,” in APA Handbook of Community Psychology: Methods for Community Research and Action for Diverse Groups and Issues, ed. M. A. Bond , I. Serrano‐García , C. B. Keys , and M. Shinn (American Psychological Association, 2017), 571–585, 10.1037/14954-034.

[20] M. E. Madyaningrum , C. C. Sonn , and A. T. Fisher , “Disability Organizations as Empowering Settings: Challenging Stigmatization, Promoting Emancipation,” American Journal of Community Psychology 69, no. 3–4 (2022): 474–483, 10.1002/ajcp.12560. 34633668

[21] S. P. Segal , C. Silverman , and T. Temkin , “Empowerment and Self‐Help Agency Practice for People With Mental Disabilities,” Social Work 38, no. 6 (1993): 705–712, 10.1093/sw/38.6.705. PMC7575187 8256142

[22] T. Niesz , L. Koch , and P. D. Rumrill , “The Empowerment of People With Disabilities Through Qualitative Research,” Work (Reading, Mass.) 31, no. 1 (2008): 113–125, 10.3233/WOR-2008-00793. 18820426

[23] L. Davy , “Between an Ethic of Care and an Ethic of Autonomy: Negotiating Relational Autonomy, Disability, and Dependency,” Angelaki 24, no. 3 (2019): 101–114, 10.1080/0969725X.2019.1620461.

[24] C. Edwards and C. Loughnane , “‘Plenty of Disabled People Care’: Revealing Reciprocity and Interdependence in Disabled People's Everyday Caregiving Practices,” Scandinavian Journal of Disability Research 26, no. 1 (2024): 588–600, 10.16993/sjdr.1182.

[25] K. Austin , C. Pistawka , C. J. D. Ross , et al., “Empowerment in Adolescent Patients With a Disability/Chronic Condition: A Scoping Review,” Children 12, no. 1 (2025): 49, 10.3390/children12010049. PMC11764063 39857880

[26] Y. Fan , N. Ma , L. Ma , W. Xu , J. Steven Lamberti , and E. D. Caine , “A Community‐Based Peer Support Service for Persons With Severe Mental Illness in China,” BMC Psychiatry 18, no. 1 (2018): 170, 10.1186/s12888-018-1763-2. PMC5987580 29866096

[27] Y. Fan , N. Ma , L. Ma , et al., “Feasibility of Peer Support Services Among People With Severe Mental Illness in China,” BMC Psychiatry 19, no. 1 (2019): 360, 10.1186/s12888-019-2334-x. PMC6854628 31727108

[28] M. Hennink and B. N. Kaiser , “Sample Sizes for Saturation in Qualitative Research: A Systematic Review of Empirical Tests,” Social Science & Medicine (1982) 292 (2022): 114523, 10.1016/j.socscimed.2021.114523. 34785096

[29] Standardization Administration of China, General Administration of Quality Supervision, Inspection and Quarantine of the People's Republic of China , GB/T 26341‐2010: Classification and Grading Criteria of Disability [残疾人残疾分类和分级] (Standards Press of China, 2011) [in Chinese].

[30] K. Charmaz , Constructing Grounded Theory: A Practical Guide Through Qualitative Analysis (Sage, 2006).

[31] J. Rappaport , “In Praise of Paradox: A Social Policy of Empowerment Over Prevention,” American Journal of Community Psychology 9, no. 1 (1981): 1–25, 10.1007/BF00896357. 7223726

[32] J. Lord and P. Hutchison , “The Process of Empowerment: Implications for Theory and Practice,” Canadian Journal of Community Mental Health 12, no. 1 (1993): 5–22, 10.7870/cjcmh-1993-0001.

[33] W. J. A. Wright , C. Howdle , N. S. Coulson , and A. De Simoni , “Exploring the Types of Social Support Exchanged by Survivors of Pediatric Stroke and Their Families in an Online Peer Support Community: Qualitative Thematic Analysis,” Journal of Medical Internet Research 26 (2024): e49440, 10.2196/49440. PMC10980984 38488858

[34] M. Tistad , L. Hultman , A. Wohlin Wottrich , and L. von Koch , “The Lived Experience of Participating in Online Peer‐to‐Peer Groups After Acquired Brain Injury: Phenomenological Study,” Journal of Medical Internet Research 27 (2025): e67658, 10.2196/67658. PMC11979533 40131323

[35] A. Bandura , Social Learning Theory (Prentice‐Hall, 1977).

[36] T. Borkman , “Experiential Knowledge: A New Concept for the Analysis of Self‐Help Groups,” Social Service Review 50, no. 3 (1976): 445–456, 10.1086/643401.

[37] S. Danforth , “Independence, Dependence, and Intellectual Disability: From Cultural Origins to Useful Application,” Philosophical Inquiry in Education 28, no. 2 (2021): 95–109, 10.7202/1082918ar.

[38] J. A. Bourke , “The Lived Experience of Interdependence: Support Worker Relationships and Implications for Wider Rehabilitation,” Brain Impairment 23, no. 1 (2022): 118–124, 10.1017/BrImp.2021.24.

[39] J. Hammel , S. Magasi , A. Heinemann , et al., “Environmental Barriers and Supports to Everyday Participation: A Qualitative Insider Perspective From People With Disabilities,” Archives of Physical Medicine and Rehabilitation 96, no. 4 (2015): 578–588, 10.1016/j.apmr.2014.12.008. 25813890

[40] R. Mpango , J. Kalha , D. Shamba , et al., “Challenges to Peer Support in Low‐ and Middle‐Income Countries During Covid‐19,” Globalization and Health 16, no. 1 (2020): 90, 10.1186/s12992-020-00622-y. PMC7517058 32977816

[41] I. H. B. Bennwik , I. Oterholm , and B. Kelly , “‘My Disability Was My Own Responsibility’: An Institutional Ethnography of the Transitional Experiences of Disabled Young People Leaving Care,” Children and Youth Services Review 146 (2023): 106813, 10.1016/j.childyouth.2023.106813.

[42] T. McAuliffe , B. Bermingham , and H. Bourke‐Taylor , “The Rewards and Challenges of Self‐Managing National Disability Insurance Scheme: Exploration of Parental Perspectives,” Australian Occupational Therapy Journal 72, no. 1 (2025): e13007, 10.1111/1440-1630.13007. 39743491

