# Supplementary material for: Relational Empowerment in Peer‐Mediated Rehabilitation for People With Disabilities: A Constructivist Grounded Theory Study
Source: Health Expect. 2026 Nov 6;29(6):e70966. doi: 10.1111/hex.70966 (PMC13742376; doi:10.1111/hex.70966)
Supplement: Supplementary file 1 [file HEX-29-e70966-s001.docx]

**Supplementary file 1**

**Title:** Relational Empowerment in Peer-Mediated Rehabilitation for People with Disabilities: A Constructivist Grounded Theory Study

**Table S1.** Consolidated criteria for reporting qualitative studies (COREQ): 32-item checklist

**S2.** Semi-structured interview topic guide and iterative probes

**Table S3.** GRIPP2 short form

Table S1. Consolidated criteria for reporting qualitative studies (COREQ): 32-item checklist

| No. Item | Guide questions/description | Page Number |
| --- | --- | --- |
| **Domain 1: Research team and reﬂexivity** |  |  |
| *Personal Characteristics* |  |  |
| 1. Interviewer/facilitator | Which author/s conducted the interview or focus group? | 9 |
| 2. Credentials | What were the researcher’s credentials? E.g. PhD, MD | Title page; 12 |
| 3. Occupation | What was their occupation at the time of the study? | Title page; 12 |
| 4. Gender | Was the researcher male or female? | 9 |
| 5. Experience and training | What experience or training did the researcher have? | 12 |
| *Relationship with participants* |  |  |
| 6. Relationship established | Was a relationship established prior to study commencement? | 9 |
| 7. Participant knowledge of the interviewer | What did the participants know about the researcher? e.g. personal goals, reasons for doing the research | 12 |
| 8. Interviewer characteristics | What characteristics were reported about the interviewer/facilitator? e.g. Bias, assumptions, reasons and interests in the research topic | 12 |
| **Domain 2: study design** |  |  |
| *Theoretical framework* |  |  |
| 9. Methodological orientation and Theory | What methodological orientation was stated to underpin the study? e.g. grounded theory, discourse analysis, ethnography, phenomenology, content analysis | 7-10 |

| No. Item | Guide questions/description | Page Number |
| --- | --- | --- |
| *Participant selection* |  |  |
| 10. Sampling | How were participants selected? e.g. purposive, convenience, consecutive, snowball | 8 |
| 11. Method of approach | How were participants approached? e.g. face-to-face, telephone, mail, email | 8 |
| 12. Sample size | How many participants were in the study? | 8, 13 |
| 13. Non-participation | How many people refused to participate or dropped out? Reasons? | 8 |
| *Setting* |  |  |
| 14. Setting of data collection | Where was the data collected? e.g. home, clinic, workplace | 10 |
| 15. Presence of non-participants | Was anyone else present besides the participants and researchers? | 9 |
| 16. Description of sample | What are the important characteristics of the sample? e.g. demographic data, date | 13 |
| *Data collection* |  |  |
| 17. Interview guide | Were questions, prompts, guides provided by the authors? Was it pilot tested? | 9 |
| 18. Repeat interviews | Were repeat interviews carried out? If yes, how many? | N/A |
| 19. Audio/visual recording | Did the research use audio or visual recording to collect the data? | 10 |
| 20. Field notes | Were ﬁeld notes made during and/or after the interview or focus group? | 10 |
| 21. Duration | What was the duration of the interviews or focus group? | 10 |

| No. Item | Guide questions/description | Page Number |
| --- | --- | --- |
| 22. Data saturation | Was data saturation discussed? | 8 |
| 23. Transcripts returned | Were transcripts returned to participants for comment and/or correction? | 10, 12 |
| **Domain 3: analysis and ﬁndings** |  |  |
| *Data analysis* |  |  |
| 24. Number of data coders | How many data coders coded the data? | 10 |
| 25. Description of the coding tree | Did authors provide a description of the coding tree? | 11; Supplementary file 2 |
| 26. Derivation of themes | Were themes identiﬁed in advance or derived from the data? | 11 |
| 27. Software | What software, if applicable, was used to manage the data? | 10 |
| 28. Participant checking | Did participants provide feedback on the ﬁndings? | 2, 12 |
| *Reporting* |  |  |
| 29. Quotations presented | Were participant quotations presented to illustrate the themes/ﬁndings? Was each quotation identiﬁed? e.g. participant number | 13-27 |
| 30. Data and ﬁndings consistent | Was there consistency between the data presented and the ﬁndings? | 13-27 |
| 31. Clarity of major themes | Were major themes clearly presented in the ﬁndings? | 13-27 |
| 32. Clarity of minor themes | Is there a description of diverse cases or discussion of minor themes? | 13-27 |

S2. Semi-structured interview topic guide and iterative probes

**Opening statement**

Thank you for taking part in this interview. We are interested in your experience of participating in self-help and mutual-aid rehabilitation. We would like to understand not only what services you received, but also how participation affected your everyday life, your relationships with peers, your sense of self, and your ability to act or help others. There are no right or wrong answers. Critical, negative, or uncertain experiences are also important. You may choose not to answer any question, and all information will be treated confidentially.

**1. Life before participation**

1. Could you tell me about your life before you first participated in self-help and mutual-aid rehabilitation?

**Probes:**

- What was your daily life like at that time?
- What were the main difficulties you faced in rehabilitation, self-care, going out, communication, family life, or social participation?
- Did you feel isolated, dependent, uncertain, or unable to act in some areas of life?
- What kinds of support or information were missing at that time?
- Were there any things you could already manage by yourself before joining the service?

**2. Pathway into self-help and mutual-aid rehabilitation**

2. How did you first hear about or come into contact with this service?

**Probes:**

- Was it introduced by a peer, family member, rehabilitation professional, disability organization, community worker, or another channel?
- What made you decide to participate?
- Did you have any hesitation, resistance, or uncertainty before joining?
- Were distance, transport, cost, accessibility, or time important in your decision?
- Were there people who encouraged, pushed, accompanied, or “brought you out”?

**3. First experience of peer spaces**

3. Please recall your first experience of entering the service space or meeting other participants. What impressed you most?

**Probes:**

- What was it like to meet people with similar experiences?
- Did seeing other people with disabilities change how you understood your own life or future?
- Did you feel safe, understood, judged, uncomfortable, or distant in that space?
- Were there particular people, moments, conversations, or activities that stayed with you?
- Did you feel that peers were more credible or easier to trust than non-disabled professionals? Why or why not?

**4. Peer mirroring and recognition of possibility**

4. Did participation help you see new possibilities for yourself? If so, how did that happen?

**Probes:**

- Did any peer make you think, “Maybe I can also do this”?
- Was the peer’s disability type, age, experience, or life stage important for you?
- Were there situations where peers did not feel similar enough for you to identify with them?
- Were there differences between same-disability and cross-disability interactions?
- Did the service space help you regain confidence temporarily or repeatedly?

**5. Learning through lived experience and professional support**

5. What kinds of knowledge or skills did you learn through self-help and mutual-aid activities?

**Probes:**

- Did you learn practical skills related to rehabilitation, transfer, self-care, pressure sore prevention, catheterization, assistive devices, communication, going out, or daily living?
- What did you learn from peers? What did you learn from professionals?
- Were you able to apply what you learned in everyday life?
- Can you give a concrete example of something you learned and later used?
- Were there times when peer experience was not enough, inaccurate, or needed professional correction?
- Did you ever share your own practical experience with others?

**6. Changes in everyday life and sense of agency**

6. After participating, did anything change in how you managed daily life or saw yourself?

**Probes:**

- Did you feel more able to make decisions, solve problems, go out, communicate, or participate in activities?
- Did your relationship with family members, peers, professionals, or the community change?
- Did participation change your sense of being dependent, being a burden, being ordinary, or being valuable?
- Were the changes temporary, gradual, or lasting?
- Were there areas where little changed?

**7. From receiving help to helping others**

7. Have you ever moved from mainly receiving help to helping, teaching, encouraging, or guiding others?

**Probes:**

- What kind of help did you provide?
- Did you become a peer mentor, assistant teacher, volunteer, group leader, informal adviser, or information provider?
- What made you willing or able to help others?
- How did helping others affect your confidence, identity, sense of value, or responsibility?
- Did helping others create pressure, fatigue, or burden?
- Are there people who benefit from the service but do not want to become helpers? Why?

**8. Interruptions, limits, and reversals**

8. Empowerment does not always happen smoothly. Were there moments when participation was difficult, disappointing, interrupted, or less useful than expected?

**Probes:**

- What barriers limited your participation or change?
- Did limited publicity, accessibility, transport, cost, service resources, age limits, family arrangements, or professional knowledge gaps affect you?
- Did cross-disability differences make mutual aid difficult?
- Were there times when you felt you moved backward, lost confidence, or could not continue?
- Who might be excluded or difficult to reach through the current service model?
- What kinds of support would make the process more sustainable?

**9. Public expression and service demands**

9. Based on your experience, what would you like service providers, communities, or policymakers to understand?

**Probes:**

- What changes are needed in publicity, referral, accessibility, assistive devices, professional support, or service design?
- Have you ever shared suggestions with staff, peers, organizations, or public platforms?
- Do you think participants should be involved in improving the service? In what way?
- What kinds of needs are still not heard or not responded to?

**10. Overall reflection**

10. Looking back, how would you describe your overall journey of participation?

**Probes:**

- What kind of person were you before participating, and how do you see yourself now?
- Which part of the process was most important for you?
- If you were introducing this service to someone who is still at home or hesitant to join, what would you say?
- Is there anything important that we have not asked?

**Closing statement**

Thank you for sharing your experience. Your account will help us better understand how self-help and mutual-aid rehabilitation works in everyday life, including its strengths, limits, and areas for improvement.

**Table S3.** GRIPP2 short form

| Section and topic | Item | Reported on page No |
| --- | --- | --- |
| 1: Aim | Report the aim of PPI in the study | Title page; 2 |
| 2: Methods | Provide a clear description of the methods used for PPI in the study | 2, 12 |
| 3: Study results | Outcomes—Report the results of PPI in the study, including both positive and negative outcomes | 12 |
| 4: Discussion and conclusions | Outcomes—Comment on the extent to which PPI influenced the study overall. Describe positive and negative effects | 2, 12 |
| 5: Reflections/critical perspective | Comment critically on the study, reflecting on the things that went well and those that did not, so others can learn from this experience | 2, 12 |

PPI=patient and public involvement
