# Supplementary material for: Relational Empowerment in Peer‐Mediated Rehabilitation for People With Disabilities: A Constructivist Grounded Theory Study
Source: Health Expect. 2026 Nov 6;29(6):e70966. doi: 10.1111/hex.70966 (PMC13742376; doi:10.1111/hex.70966)
Supplement: Supplementary file 2 [file HEX-29-e70966-s002.docx]

**Supplementary file 2. Supplementary coding framework and analytic audit trail**

**Title:** Relational Empowerment in Peer-Mediated Rehabilitation for People with Disabilities: A Constructivist Grounded Theory Study

**Table S1.** Analytic procedure used in the constructivist grounded theory analysis

**Table S2.** Final coding framework, supporting evidence, and analytic boundaries

**Table S3.** Negative and limiting cases retained during analysis

**Table S4.** Key methodological decisions relevant to interpretation

**Supplementary note.** Materials retained internally but not submitted

This supplementary file provides a concise evidence trail for the constructivist grounded theory analysis. It reports the analytic procedures, final coding framework, negative cases, and key methodological decisions needed to assess transparency and credibility. Segment identifiers are retained to show how analytic claims can be traced within the de-identified internal audit dataset. In this study, initial coding was deliberately fine-grained and allowed multiple code applications to the same segment. Such counts are useful for internal audit but can be misleading if interpreted as the number of substantive concepts. The submitted evidence therefore emphasizes coding logic, category development, negative cases, and traceability rather than volume of coded fragments.

**Table S1. Analytic procedure used in the constructivist grounded theory analysis**

| **Analytic stage** | **What was done** | **Evidence retained for audit** | **Relevance for this study** |
| --- | --- | --- | --- |
| Data preparation | Interview and focus group transcripts were de-identified and organized at line or meaning-unit level. Each analytic segment was assigned a unique identifier. | De-identified segment table retained internally. | Preserved traceability without disclosing full participant narratives. |
| Initial coding | Segments were coded closely to the data. Codes were written, where possible, as actions or processes; in vivo expressions were retained when analytically useful. Multiple codes could be assigned to one segment. | Initial codebook and coded segment records retained internally. | Captured processes such as being introduced to peer spaces, learning embodied skills, reinterpreting self, and helping others. |
| Constant comparison | Segments, participants, codes, categories, and negative cases were compared throughout analysis. | Comparison notes and analytic memos retained internally. | Helped distinguish similar concepts, such as peer mirroring versus emotional support, and experiential knowledge versus professional guidance. |
| Focused coding | Initial codes with stronger explanatory relevance were grouped, refined, or separated. | Focused coding crosswalk retained internally. | Connected detailed initial codes to higher-level categories used in the Results section. |
| Category development | Focused codes were developed into categories by examining properties, dimensions, conditions, consequences, and boundaries. | Category table and evidence map retained internally. | Produced the final category structure and clarified each category's analytic boundary. |
| Theoretical integration | Categories were examined in relation to one another to develop a process-oriented explanation of empowerment. | Theory map retained internally and summarized below. | Supported the core process of regaining agency through peer mutual aid. |
| Negative case analysis | Contradictory, limiting, and less positive cases were retained and compared against emerging categories. | Negative case table retained internally and summarized below. | Prevented an overly linear or uniformly positive interpretation of self-help and mutual aid. |

**Table S2. Final coding framework, supporting evidence, and analytic boundaries**

| **Category** | **Analytic role in the process model** | **Focused concepts retained** | **Evidence links** | **Analytic boundary** |
| --- | --- | --- | --- | --- |
| C1. Disempowerment as a starting point: shrinking life worlds and fractured self-understanding | Describes the pre-participation condition from which empowerment became meaningful. | Social withdrawal; internalized stigma; seeking a way out of a narrowed life world. | P07_U013; P07_U014; P12_U002; P10_U001; P01_U007. | Not all non-participation was caused by stigma or isolation; perceived mismatch between service content and personal needs also mattered. |
| C2. Entering peer spaces: referral, being brought out, and gradual participation | Explains how participants became connected to self-help and mutual-aid rehabilitation spaces. | Peer encountering; graduated engagement; entering public space; orientation to the platform and its activities. | P01_U001; P02_U003; P12_U003; P03_U007. | Entry was often mediated by professionals, organizations, peers, or family members; it should not be written as purely self-initiated or linear. |
| C3. Peer mirroring and safe spaces: seeing possibility through similar others | Identifies the reflective mechanism through which participants reimagined what was possible. | Peer mirroring; emotional resonance; safe space experience; recognition; seeing peers as credible teachers. | P01_U008; P02_U025; P03_U036; P01_U030; P01_U016; P04_U010. | Peer similarity strengthened credibility, but mutual understanding across disability categories was not automatic. |
| C4. Experiential reciprocity: translating lived knowledge into practical capacity | Explains how lived experience became usable knowledge for rehabilitation, self-management, and daily life. | Experiential knowledge sharing; instrumental mutual aid; skill acquisition; self-efficacy enhancement; translating experience into self-management. | P01_U003; P05_U024; P05_U047; P10_U002; P20_U013; P03_U009; P16_U012; P16_U015; P02_U011. | Peer experience was empowering when practical and credible, but it required professional calibration and contextual judgment. |
| C5. Role transformation: from receiving help to helping others | Represents a key empowerment outcome and feedback loop in which participants became resources for others. | Role expansion; role-based identity change; mutual healing; converting disability experience into a helping resource. | P05_U003; P01_U076; P01_U046; P09_U020; P16_U030. | Helping roles could also create fatigue and responsibility burden; role transformation requires support and recognition. |
| C6. Interruptions and reversals: conditions that shaped the empowerment process | Captures cross-cutting conditions that delayed, weakened, or redirected the empowerment process. | Non-linear empowerment; limited publicity and service reach; accessibility barriers; uneven district resources; professional knowledge gaps; daily support arrangements; cross-disability fit. | P01_U023; P04_U027; P10_U003; P13_U003; P01_U061. | Barriers were situated rather than universal; some participants experienced transport or access as manageable. |
| C7. Public expression and service demands as a tentative extension | Describes emerging service voice beyond individual change. | Service suggestions; policy and service information needs; assistive device information; service design feedback; cautious use of collective advocacy concepts. | P01_U024; P01_U040; P10_U024; P13_U019;  P04_U017. | Evidence supported service demands and public expression, but not strong claims of organized collective advocacy. |

Note: Evidence links are illustrative examples of de-identified segment identifiers drawn from the internal audit dataset; they are not an exhaustive list of all segments supporting each category.

**Table S3. Negative and limiting cases retained during analysis**

| **Case ID** | **Related category** | **Contradictory or limiting evidence** | **Analytic implication** |
| --- | --- | --- | --- |
| NC_P03_U023 | C1/C2 | A participant refused invitations for several years because he believed he already knew the training skills and did not see the need to attend. | Non-participation should not be reduced to stigma, fear, or lack of access; perceived relevance matters. |
| NC_P01_U012 | C6 | A participant reported that local public transport was relatively convenient and did not constitute a major barrier for him. | Accessibility barriers should be treated as situated conditions rather than universal experience. |
| NC_P01_U053 and NC_P06_U003 | C4/C6 | Participants questioned the professional adequacy of some peer or staff knowledge, including wheelchair-related knowledge and caregiver experience. | Experiential knowledge is empowering when practical and credible, but it needs professional support and contextual judgment. |
| NC_P01_U061 and NC_P01_U066 | C3/C6 | A participant emphasized that different disability groups may not easily understand each other's embodied needs or social experiences. | Peer similarity is part of the mechanism; cross-disability mutual aid requires additional translation and mediation. |
| NC_P01_U070 | C5 | A participant described becoming a teacher as physically and emotionally tiring because he had to support many learners while managing his own disability. | Helping others can be empowering and burdensome; peer helper roles require organizational support. |

**Table S4. Key methodological decisions relevant to interpretation**

| **Issue** | **Decision used in this study** | **Rationale** | **How this is reflected in reporting** |
| --- | --- | --- | --- |
| Reporting of coding volume | Raw counts of meaning units and initial code applications were retained internally but not presented as indicators of analytic strength. | Fine-grained coding and multiple coding make these counts methodologically uninformative if interpreted as substantive concepts. | The supplement reports analytic procedures, category evidence, and negative cases rather than raw coding volume. |
| Grounded theory procedure | The analysis followed constructivist grounded theory procedures: initial coding, focused coding, constant comparison, memo-writing, category development, and theoretical integration. | This maintained methodological coherence with the Charmaz approach. | The supplement does not present a Strauss-style axial coding matrix or paradigm model. |
| Use of empowerment theory | Empowerment theory was used as a sensitizing perspective rather than a predetermined coding framework. | This reduced the risk of forcing data into pre-existing empowerment domains. | Categories are reported as data-grounded processes before being interpreted theoretically. |
| Quotations and traceability | Formal claims were linked to de-identified segment identifiers. | This preserved auditability and reduced the risk of unsupported illustrative claims. | Category evidence is reported through Pxx_Uxxx evidence links. |
| Confidentiality | Full transcripts and complete raw coding tables were not submitted. | Detailed biographical, service-use, and contextual information could increase re-identification risk. | A concise category-evidence table and negative case table are provided instead. |

**Supplementary note: Materials retained internally but not submitted**

The research team retained the following materials as internal audit records: de-identified transcript segment table, initial coding records, focused coding crosswalk, category development table, analytic memos, negative case notes, and theory map. These materials were used to support traceability during analysis but are not included in the journal supplement because the full materials are lengthy and contain contextual details that may compromise confidentiality. The submitted supplement therefore presents the minimum evidence needed to document the analytic process, category structure, negative cases, and key methodological decisions.
